# Modelling the cascade of biomarker changes in *GRN*-related frontotemporal dementia

**DOI:** 10.1136/jnnp-2020-323541

**Published:** 2021-01-15

**Authors:** Jessica L Panman, Vikram Venkatraghavan, Emma L van der Ende, Rebecca M E Steketee, Lize C Jiskoot, Jackie M Poos, Elise G P Dopper, Lieke H H Meeter, Laura Donker Kaat, Serge A R B Rombouts, Meike W Vernooij, Anneke J A Kievit, Enrico Premi, Maura Cosseddu, Elisa Bonomi, Jaume Olives, Jonathan D Rohrer, Raquel Sánchez-Valle, Barbara Borroni, Esther E Bron, John C Van Swieten, Janne M Papma, Stefan Klein, GENFI consortium investigators, Sónia Afonso

**Affiliations:** 1 Department of Neurology, Erasmus Medical Center, Rotterdam, The Netherlands; 2 Department of Radiology, Leiden University Medical Center, Leiden, The Netherlands; 3 Department of Radiology and Nuclear Medicine, Erasmus Medical Center, Rotterdam, The Netherlands; 4 Department of Neurology, Erasmus MC, Rotterdam, The Netherlands; 5 Department of Clinical Genetics, Erasmus Medical Center, Rotterdam, The Netherlands; 6 Institute for Psychology, Leiden University, Leiden, The Netherlands; 7 Centre for Neurodegenerative Disorders, University of Brescia, Brescia, Italy; 8 Alzheimer's Disease and Other Cognitive Disorders Unit, Hospital Clínic de Barcelona, Barcelona, Spain; 9 Dementia Research Centre, UCL Institute of Neurology, London, UK; 10 Institut d’Investigacions Biomèdiques August Pi i Sunyer, Barcelona, Spain

## Abstract

**Objective:**

Progranulin-related frontotemporal dementia (FTD-*GRN*) is a fast progressive disease. Modelling the cascade of multimodal biomarker changes aids in understanding the aetiology of this disease and enables monitoring of individual mutation carriers. In this cross-sectional study, we estimated the temporal cascade of biomarker changes for FTD-*GRN*, in a data-driven way.

**Methods:**

We included 56 presymptomatic and 35 symptomatic *GRN* mutation carriers, and 35 healthy non-carriers. Selected biomarkers were neurofilament light chain (NfL), grey matter volume, white matter microstructure and cognitive domains. We used discriminative event-based modelling to infer the cascade of biomarker changes in FTD-*GRN* and estimated individual disease severity through cross-validation. We derived the biomarker cascades in non-fluent variant primary progressive aphasia (nfvPPA) and behavioural variant FTD (bvFTD) to understand the differences between these phenotypes.

**Results:**

Language functioning and NfL were the earliest abnormal biomarkers in FTD-*GRN*. White matter tracts were affected before grey matter volume, and the left hemisphere degenerated before the right. Based on individual disease severities, presymptomatic carriers could be delineated from symptomatic carriers with a sensitivity of 100% and specificity of 96.1%. The estimated disease severity strongly correlated with functional severity in nfvPPA, but not in bvFTD. In addition, the biomarker cascade in bvFTD showed more uncertainty than nfvPPA.

**Conclusion:**

Degeneration of axons and language deficits are indicated to be the earliest biomarkers in FTD-*GRN*, with bvFTD being more heterogeneous in disease progression than nfvPPA. Our data-driven model could help identify presymptomatic *GRN* mutation carriers at risk of conversion to the clinical stage.

## Introduction

Mutations in the progranulin (*GRN*) gene on chromosome 17q21 are a major cause of autosomal dominant inherited frontotemporal dementia (FTD).^1 2^ The majority of mutation carriers develops a behavioural variant FTD (bvFTD) phenotype,^3^ and another significant proportion of patients present with non-fluent variant primary progressive aphasia (nfvPPA).^3 4^ The age of symptom onset varies between 35 years and 90 years in *GRN* mutation carriers,^1 2^ without clear associations with familial age of onset.^4^ Brain changes in FTD-*GRN* patients can evolve symmetrically, or predominantly asymmetrically, in either the left or right hemisphere.^5 6^

Recent longitudinal studies have suggested that the time-window between emerging pathophysiological changes and the first clinical symptoms is short in *GRN* mutation carriers, and covers only 2–4 years.^7 8^ During this period, the serum neurofilament light chain (NfL) level—a marker of axonal degeneration—increases twofold–threefold,^9 10^ loss of grey and white matter emerges,^7 11^ and cognitive functioning declines.^8^ However, most of the biomarker studies in FTD-*GRN* have investigated one type of biomarker, that is, fluid, neuroimaging or cognition, leaving the temporal relations and ordering of these biomarkers unknown. These temporal relations could potentially provide novel insights into disease progression mechanisms in *GRN* mutation carriers. Moreover, because of the fast progression of pathophysiological changes, determining the earliest abnormal biomarker is crucial, as the optimal window of opportunity for treatment might be small.

Recently, novel data-driven methods for disease progression modelling have emerged, focusing on the cascade of biomarker changes.^12 13^ Event-based models are a class of disease progression models that estimate the cascade of biomarker changes derived from cross-sectional data.^6 13 14^ This is done without strong a priori assumptions regarding the relationship between different biomarkers. A promising novel method that estimates the cascade of biomarker change is discriminative event-based modelling (DEBM).^13 15^ This model is robust to disease phenotypic heterogeneity in a cohort and can handle missing data.

In this study, we use DEBM to estimate the temporal cascade of biomarker changes in presymptomatic and symptomatic FTD-*GRN* mutation carriers, distinguishing between early and late biomarkers. Furthermore, we determine phenotypic differences in patterns of biomarker changes in nfvPPA and bvFTD, to gain more insights into their distinct disease progression mechanisms.

## Methods

### Sample and study procedures

Subjects were recruited prospectively from three European centres of the Genetic Frontotemporal dementia Initiative (GENFI): Rotterdam (the Netherlands), Brescia (Italy) and Barcelona (Spain). We collected cognitive and clinical data, MRI and serum samples from 126 participants. We included 35 symptomatic *GRN* mutation carriers (Rotterdam: n=11, Brescia: n=22, and Barcelona: n=2), 56 presymptomatic *GRN* mutation carriers (Rotterdam: n=33, Brescia: n=17, and Barcelona: n=6) and 35 cognitively healthy non-carriers (Rotterdam: n=34, Brescia: n=0, and Barcelona: n=1). Local clinical genetics departments performed DNA genotyping to confirm the presence of a *GRN* mutation. Non-carriers were first-degree family members of *GRN* patients without a mutation. Symptomatic mutation carriers were diagnosed based on the established clinical criteria for bvFTD^16^ (n=17), nfvPPA^17^ (n=16) or cortico-basal syndrome^18^ (n=2). Mutation carriers were defined as presymptomatic when clinical criteria were not fulfilled, that is, behavioural or cognitive symptoms were absent.^19^ Clinical questionnaires were administered to the caregiver, spouse or a family member, that is, the Frontotemporal Lobar Degeneration Clinical Dementia Rating Scale Sum of Boxes (FTD-CDR-SOB),^20^ the Neuropsychiatric Inventory (NPI)^21^ and the Frontotemporal Dementia Rating Scale (FRS).^22^ The study was carried out according to the Declaration of Helsinki, approved by the local medical ethics board at each site, and all participants provided written informed consent.

### Biomarker collection and processing

#### Biomarker selection

For biomarker selection, we performed a literature search using Pubmed. We included studies that (1) performed research in presymptomatic *GRN* mutation carriers and (2) biomarker studies that examined biomarkers in blood or cerebrospinal fluid (CSF), neuroimaging biomarkers and cognition. We selected serum NfL,^9^ Mini Mental State Examiation (MMSE), cognitive domains of attention and processing speed, executive functioning, language and social cognition^8 23^; left and right grey matter volumes of the insula, frontal lobe, parietal lobe and temporal lobe^7 11^; and left and right white matter tracts of the anterior thalamic radiation, superior longitudinal fasciculus, uncinate fasciculus and the forceps minor.^7 24^ For detailed information about the literature review and subsequent biomarker selection, please see online supplemental appendix A.

10.1136/jnnp-2020-323541.supp1 Supplementary data

#### Neurofilament light chain

Serum samples were obtained through venepunctures and analysed with single molecular assay technology, as described previously.^10^ Samples were measured in a single laboratory, in duplicate, with an intra-assay coefficient of variation below 5%. Inter-assay variation between batches was below 8%. NfL concentrations were expressed in pg/mL.

#### MRI

Three-dimensional T1-weighted and diffusion tensor imaging were acquired with 3T MRI scanners across the three sites. MRI was missing in 25 participants due to unavailability (n=16) and insufficient quality due to motion artefacts (n=9). Availability of MRI and an overview of the scanning protocols are listed in online supplemental appendix A, Table A.1. Image processing was carried out in FMRIB Software Library (FSL),^25^ using default pipelines for grey matter volumes and white matter tracts. For grey matter volumetric regions of interest (ROI), we used the Montreal Neurological Institute atlas,^26^ and for the fractional anisotropy of white matter tracts, we used the Johns Hopkins University atlas.^27^ Left and right regions and tracts were considered separately. Raw regional volumes and fractional anisotropy values were transformed to z-scores, based on the mean and SD from the non-carriers. A detailed description of processing and ROI calculation is reported in online supplemental appendix A.

#### Cognitive assessment

Cognitive data were collected from all participants in four cognitive domains, described in detail in online supplemental appendix A. Raw cognitive test scores were transformed to z-scores based on the mean and SD in non-carriers, and then combined into cognitive domain scores similar to previous studies.^8^

#### Confounding factors correction

All selected biomarkers were tested for normality (see online supplemental appendix A for details) and log-transformed in case of a skewed distribution. As most non-carriers originated from one centre, we used presymptomatic subjects for regressing out possible confounding effects using multiple linear regression, before continuing with event-based modelling. NfL levels were corrected for age and sex. Grey matter volumes and fractional anisotropy values were corrected for age, sex, total intracranial volume and MRI scanning protocol. Cognitive domain scores were corrected for confounding effects of age, sex and total years of education.

### Temporal cascade of biomarker changes

The DEBM model introduced by Venkatraghavan *et al* 13 15 estimates the cascade of biomarker changes in a three-step process. For each biomarker, it first estimates the distributions of normal and pathological (or abnormal) values using Gaussian mixture modelling (GMM), and uses these to compute, for each subject, the probability that the biomarker is abnormal (explained in detail in online supplemental appendix B). The method then estimates the biomarker cascade independently for each subject based on the biomarker values present for that subject. The mean cascade is estimated such that the sum of the probabilistic Kendall’s Tau distances is minimised between the mean cascade and all the subject-specific cascades. For subjects with missing biomarker values, only the corresponding subset of the biomarker cascade present in the subject-specific cascade is used to compute the probabilistic Kendall’s Tau distance. Lastly, the severity of disease as a summary measure for each subject is computed by estimating the subject’s progression along the resulting disease progression timeline. In this section, we describe the experiments we performed for estimating the cascade of biomarker changes for non-imaging biomarkers, as well as for neuroimaging and non-imaging biomarkers together.

10.1136/jnnp-2020-323541.supp2 Supplementary data

#### DEBM model for non-imaging biomarkers

As imaging was missing in a lot of subjects (n=25), we first estimated the cascade of biomarker changes procedure with solely NfL and cognitive biomarkers. Since the non-carriers are healthy in this cohort, the normal Gaussians were fixed at the mean and SD of the biomarker values of the non-carriers. We used GMM only to estimate the abnormal Gaussian and the mixing parameter for each biomarker. In order to estimate the positional variance in the estimated cascade, the entire dataset was randomly sampled using bootstrap sampling with 100 different random seeds, and the cascade of biomarker change was estimated for each of those randomly sampled datasets.^13 15^

#### DEBM model for neuroimaging and non-imaging biomarkers together

For the imaging biomarkers, we modified the GMM step in DEBM to make it better suited for the FTD-*GRN* population, known for its asymmetric pattern of atrophy.^5^ Abnormal values of biomarkers that typically become abnormal late in the disease are usually under-represented in a specific patient population as compared with the early biomarkers. This could make the GMM of late biomarkers unstable, as previously reported.^15^ Due to the asymmetrical atrophy patterns of FTD-*GRN*,^5 6^ lateralised neuroimaging biomarkers that become abnormal early in the disease process may have a corresponding biomarker from the other hemisphere that remains stable until much later in the disease process. To exploit this, we assumed that the normal and abnormal Gaussians from the left and right hemispheric biomarkers (expressed as z-scores) are the same, and the biomarkers from both hemispheres only differ in their position along the disease progression timeline. With this assumption, we proposed a novel modification to the GMM optimisation called Siamese GMM, in which the biomarkers of the same region from left and right hemispheres are jointly optimised. The abnormal and normal Gaussians are shared between the left and right hemispheres, but the mixing parameters are independently estimated (see online supplemental appendix B for details). In this way, the numerical stability of GMM optimisation in the late neuroimaging biomarkers improved.

For non-imaging biomarkers, GMM was performed as described in the previous section. After GMM, further steps of DEBM modelling were carried out as usual, to estimate the complete cascade of neuroimaging and non-imaging biomarker changes in presymptomatic and symptomatic *GRN* mutation carriers. The positional variance in the estimated cascade was again estimated using bootstrap sampling with 100 different random seeds. For brevity, in the remainder of the paper, we refer to this model, which integrates neuroimaging and non-imaging biomarkers, as the multimodal DEBM.

#### Validation

To validate the DEBM models, we used 10-fold cross-validation. In each fold of the cross-validation, the DEBM model was built in the training set and the disease severity was estimated in the test set. We distinguished symptomatic mutation carriers from presymptomatic mutation carriers, and reported the corresponding sensitivity and specificity. Furthermore, in bvFTD and nfvPPA subjects, the estimated disease severity was correlated with years since symptom onset and FTD-CDR-SOB scores, using Pearson’s correlation. Symptomatic carriers without imaging biomarkers were excluded for the validation of the multimodal DEBM but were included in the non-imaging DEBM.

### Differential phenotype analysis

In order to examine the differences between bvFTD and nfvPPA variants of FTD-*GRN*, we built separate DEBM models. Presymptomatic subjects were excluded from this analysis as no phenotype information is available. The numbers of symptomatic subjects in each group (17 with bvFTD and 16 with nfvPPA) are too small to build complete DEBM models reliably. As a solution, we assumed that the biomarkers for the two phenotypes shared the same normal and abnormal biomarker distributions, and that they only differ in their position along the disease progression timeline. We hence optimised the GMM such that the normal and abnormal Gaussians were estimated without considering the phenotypes, whereas the mixing parameters were estimated separately for each phenotype. As before, we estimated the cascade of biomarker changes in the two phenotypes for non-imaging and multimodal (neuroimaging and non-imaging together) biomarkers.

## Results

### Sample

A total of 126 subjects were included in this study. Availability and characteristics of the data are listed in table 1. Details on biomarker availability and characteristics can be found in online supplemental appendix, Tables A.2 and A.3. Symptomatic mutation carriers were older, had fewer years of education and had higher scores on the NPI and FTD-CDR-SOB, and lower scores on the FRS than both presymptomatic mutation carriers and non-carriers. There were no differences in demographic or clinical characteristics between presymptomatic mutation carriers and non-carriers.

**Table 1:** Data availability and characteristics

	Symptomatic			Presymptomatic	Non-carriers
	Total	bvFTD	nfvPPA		
N					
Subjects (% female)	35* (60.0%)	17 (47.1%)	16 (75%)	56 (69.6%)	35 (54.4%)
Rotterdam	11	8	3	33	34
Brescia	22*	9	11	17	0
Barcelona	2	0	2	6	1
Data availability					
Serum NfL	91.7%	88.9%	93.8%	69.64%	91.67%
Cognitive assessment	91.7%	88.9%	93.8%	98.21%	91.67%
T1-weighted MRI	44.4%	38.9%	50.0%	96.4%	88.6%
DTI	50.0%	44.4%	56.3%	92.9%	91.4%
Sample characteristics					
Age (years)	62.57±6.72†	62.93±6.11‡	61.78±7.78§	51.52±11.42	55.15±12.55
Education (years)	10.61±4.59†	10.27±4.91‡	11.79±4.02	13.79±3.27	13.21±2.84
TIV (litres)	1.44±0.17	1.50±0.17	1.42±0.14	1.39±0.15	1.40±0.14
NPI	23.77±28.38†	28.90±30.64‡,¶	6.67±6.03¶	1.87±3.37	2.24±4.32
FRS	56.50±30.43†	48.86±29.91‡	67.20±30.96§	97.27±10.11	95.47±7.45
FTD-CDR-SOB	7.64±6.52†	9.68±7.47‡,¶	5.25±4.37§,¶	0.04±0.21	0.00±0.00
Disease duration (years)	2.45±2.01	2.37±1.92	2.48±2.29	N/A	N/A

*The two remaining patients presented with cortico-basal syndrome.

†Significant difference between symptomatic carriers and presymptomatic carriers as well as non-carriers.

‡Significant difference between bvFTD patients and presymptomatic patients as well as non-carriers.

§Significant difference between nfvPPA patients and presymptomatic patients as well as non-carriers.

¶Significant difference between bvFTD patients and nfvPPA patients.

bvFTD, behavioural variant frontotemporal dementia; DTI, diffusion tensor imaging; FRS, Frontotemporal Dementia Rating Scale; FTD-CDR-SOB, Frontotemporal Lobar Degeneration Clinical Dementia Rating Scale Sum of Boxes; mean±SD. GM, grey matter; NfL, neurofilament light chain; nfvPPA, non-fluent variant primary progressive aphasia; NPI, Neuropsychiatric Inventory; TIV, total intracranial volume.

### Cascade of biomarker changes

### Non-imaging and multimodal DEBM models

**Figure 1:**
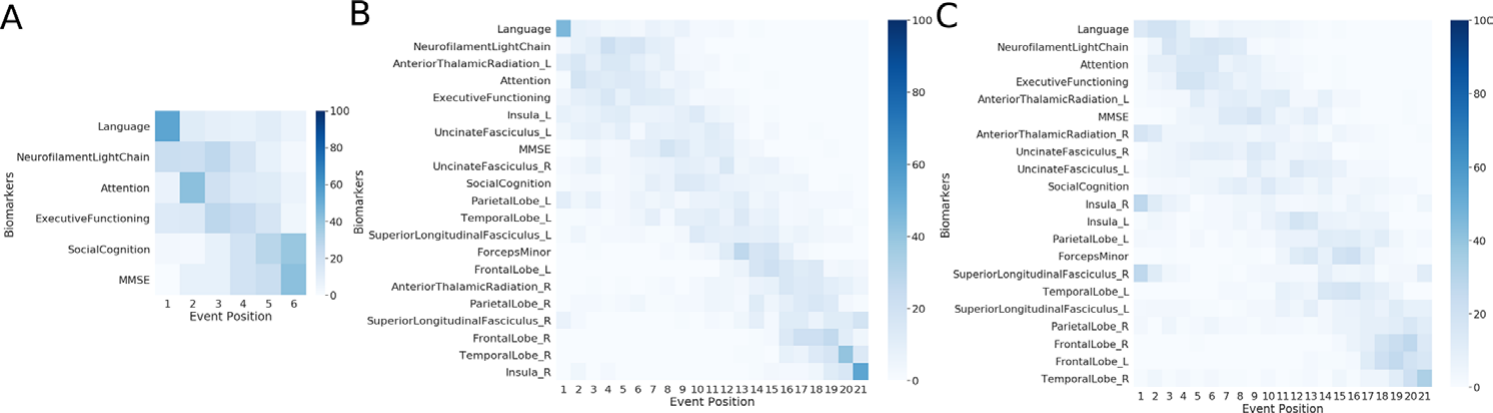
Cascade of biomarker changes in FTD-*GRN* along with the uncertainty associated with it. (A) Non-imaging biomarkers. (B) Multimodal biomarkers with Siamese GMM. (C) Multimodal biomarkers without Siamese GMM. The biomarkers are ordered based on the position in the estimated cascade. The colour map is based on the number of times a biomarker is at a position in 100 repetitions of bootstrapping. FTD-*GRN*, progranulin-related frontotemporal dementia; GMM, Gaussian mixture modelling.

**Figure 2:**
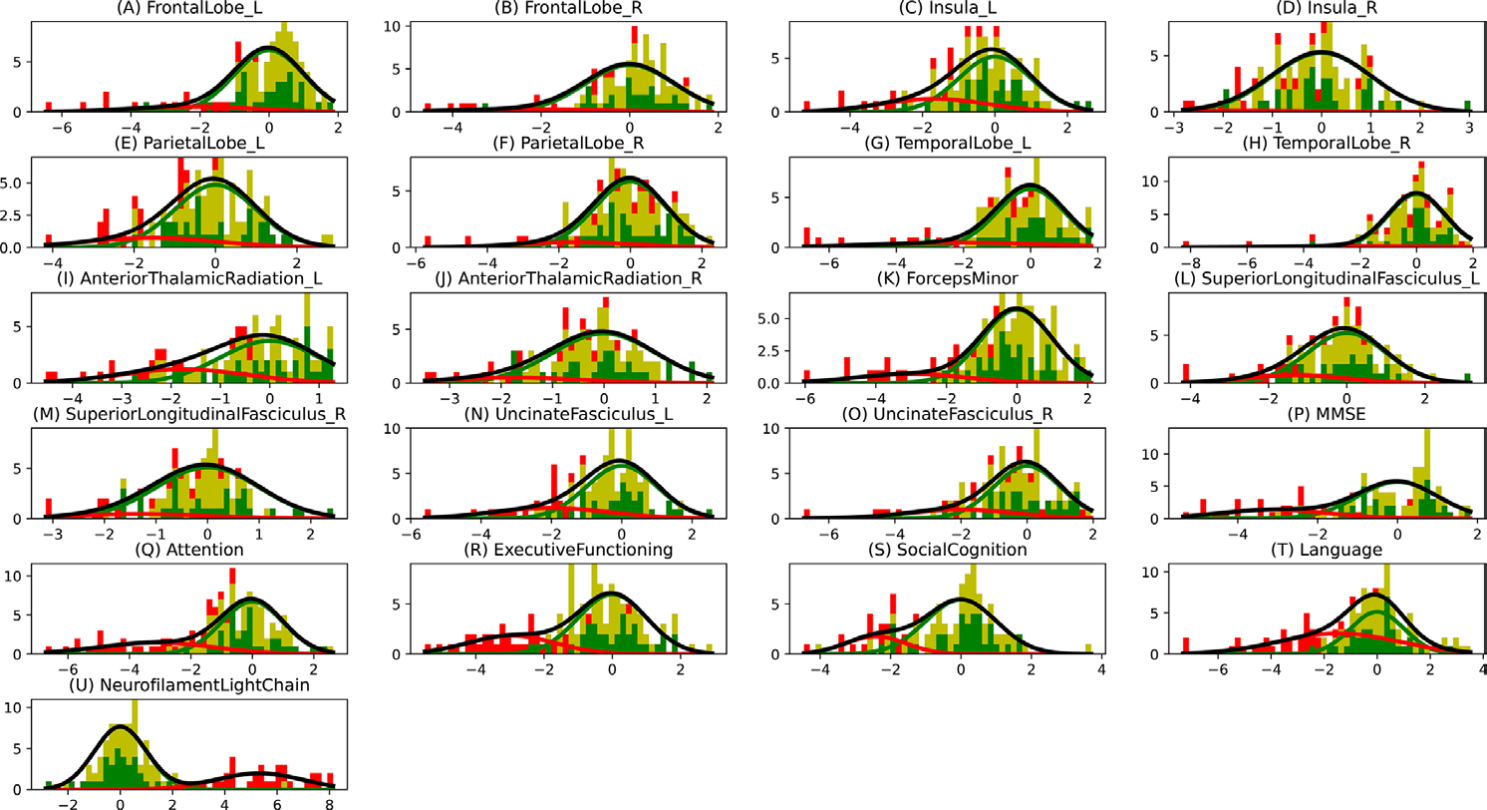
Gaussian mixture modelling (GMM) distributions. The histogram bins are divided in three colours, where the green part shows the proportion of non-carriers, the yellow part shows the proportion of presymptomatic carriers and the red part shows the proportion of symptomatic carriers. The Gaussians shown here are the ones that were estimated using GMM, where the green Gaussian is the normal one estimated using non-carriers and the red Gaussian is the abnormal one estimated using the carriers. The amplitudes of these Gaussians are based on the estimated mixing parameter. The grey curve shows the total estimated distribution, which is the summation of green and red Gaussians.

In figure 1A, B, we show the estimated mean cascade of biomarker changes and the uncertainty within the model for non-imaging and multimodal biomarkers. Language was the earliest biomarker to become abnormal, followed by NfL. It can be seen in figure 1B that left anterior thalamic radiation, left insula and bilateral uncinate fasciculi were the earliest imaging biomarkers. It can also be observed that imaging biomarkers from the left hemisphere became abnormal earlier than their right counterpart. GMM estimations with normal and abnormal Gaussian distributions are shown in figure 2, where the estimated Gaussians are seen to fit the observed histograms well. Figure 1C shows the positional variance of the cascade of multimodal biomarker changes obtained when GMM of the imaging biomarkers was done without using Siamese GMM. Generally, the positional variance was smaller with Siamese GMM than without.

### Validation

**Figure 3:**
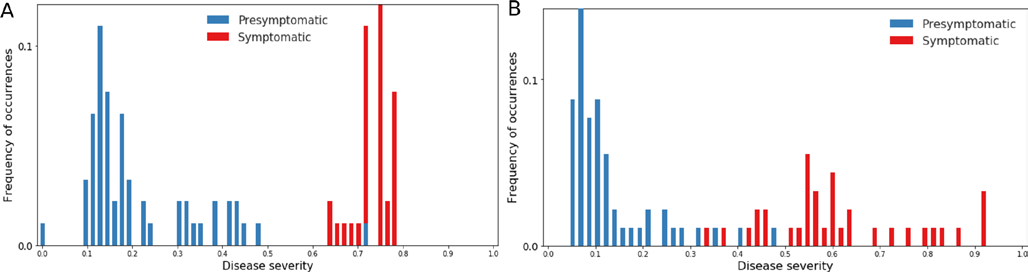
Frequency of occurrence of subjects with different disease severities, estimated using cross-validation. (A) Results using non-imaging biomarkers in discriminative event-based modelling (DEBM). (B) Results using multimodal biomarkers in DEBM.

Figure 3A, B shows the estimated disease severity when using non-imaging and multimodal biomarkers, respectively. It can be seen that estimated disease severity delineated the symptomatic subjects from the presymptomatic subjects. The sensitivity and specificity of this delineation were 1.0 and 0.982, respectively, while using non-imaging biomarkers, and 1.0 and 0.961, respectively, while using multimodal biomarkers.

**Figure 4:**
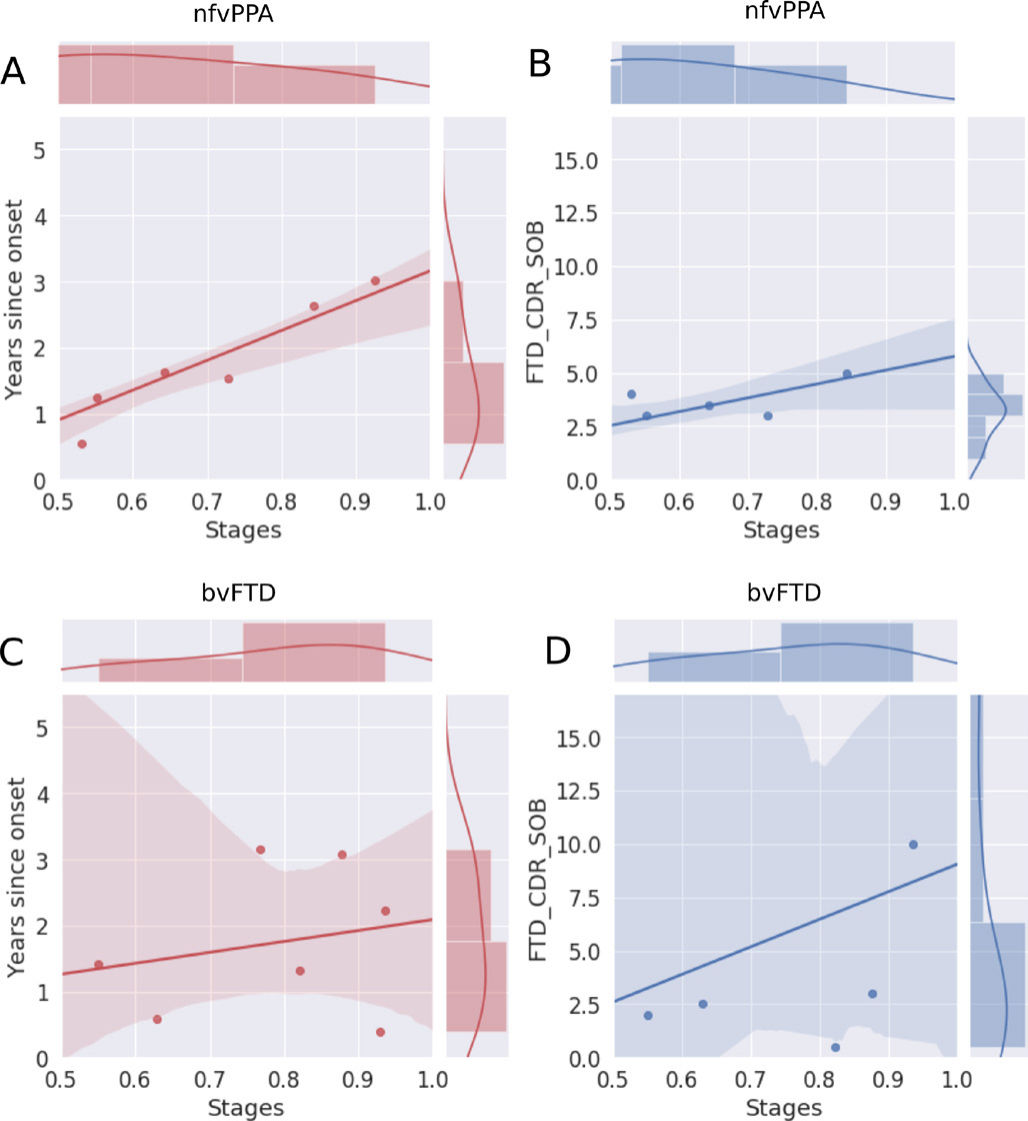
Correlation of disease severity (as estimated by multimodal DEBM using cross-validation) with years since onset and FTD-CDR-SOB. The 2D scatter plots in subfigures A and C show the correlations of disease severity with years since onset, for symptomatic nfvPPA and bvFTD subjects, respectively. The 2D scatter plot in subfigures B and D show the correlations of disease severity with FTD-CDR-SOB. The plot on top of each subfigure shows the probability density function of the disease stages. The plots on the right of subfigures A and C show the probability density functions of years since symptom onset. The plots on the right of subfigures B and D show the probability density function of FTD-CDR-SOB. 2D, two-dimensional; bvFTD, behavioural variant frontotemporal dementia; DEBM, discriminative event-based modelling; FTD-CDR-SOB, Frontotemporal Lobar Degeneration Clinical Dementia Rating Scale Sum of Boxes; nfvPPA, non-fluent variant primary progressive aphasia.

Figure 4 shows the correlation of the estimated disease severity with years since symptom onset and FTD-CDR-SB for nfvPPA and bvFTD subjects, when using multimodal DEBM. It can be seen from figure 4 that estimated disease severity strongly correlated with years since symptom onset (R=0.95, p=0.0003) and the FTD-CDR-SB (R=0.84, p=0.0189) in nfvPPA patients. However, estimated disease severity correlated poorly with years since symptom onset (R=0.22, p=0.6331) and the FTD-CDR-SB (R=0.28, p=0.5866) in bvFTD patients. Online supplemental figure B.2 shows a similar plot when using non-imaging biomarkers, where estimated disease severity did not correlate with years since symptom onset and FTD-CDR-SB, neither for nfvPPA nor for bvFTD subjects.

### Differential phenotype analysis

**Figure 5:**
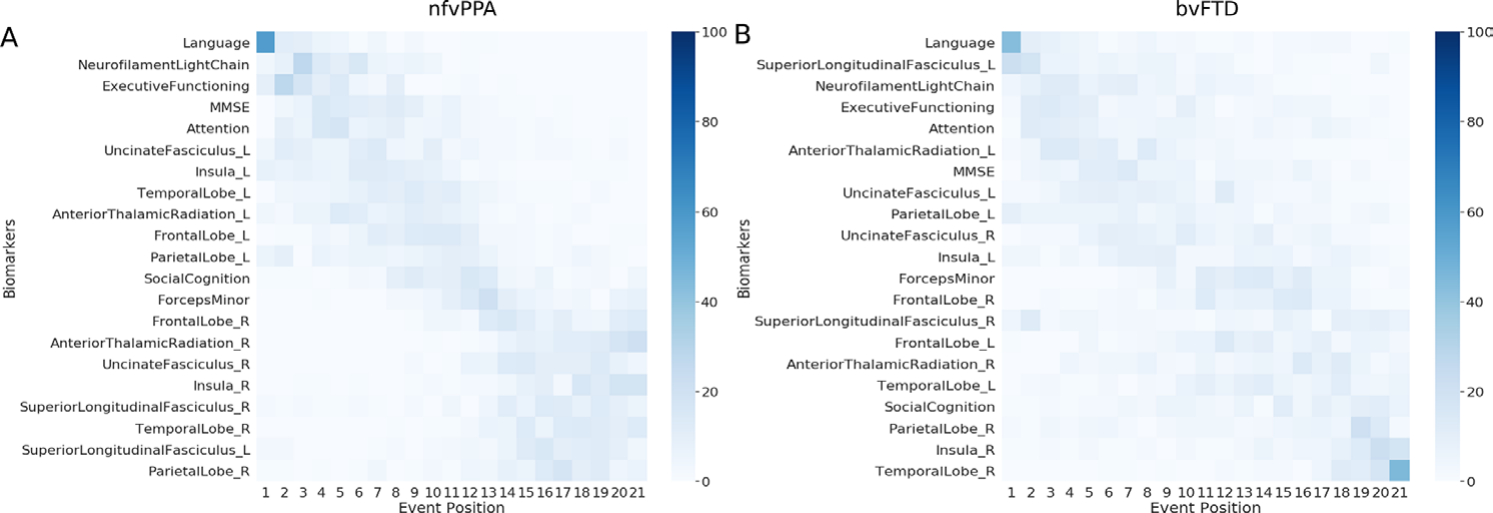
Cascade of multimodal biomarker changes in nfvPPA (A) and bvFTD (B) subjects along with the uncertainty associated with it. The biomarkers are ordered based on the position in the estimated cascade. The colour map is based on the number of times a biomarker is at a position in 100 repetitions of bootstrapping. bvFTD, behavioural variant frontotemporal dementia; nfvPPA, non-fluent variant primary progressive aphasia.

Figure 5 shows the multimodal biomarker cascade for nfvPPA and bvFTD phenotypes. nfvPPA patients showed language and NfL as first abnormal biomarkers followed by other cognitive domains. Left hemispheric imaging biomarkers became abnormal before right hemispheric imaging biomarkers, starting with the uncinated fasciculus (white matter integrity), insula and temporal lobe (grey matter volume). Only the left superior longitudinal fasciculus was estimated as late biomarker, even later then its right-sided counterpart.

Interestingly, in bvFTD patients, the biomarker ordering also indicated that language and NfL were the earliest abnormal biomarkers. In contrast to the nfvPPA, the left superior longitudinal fasciculus (white matter integrity) was estimated as the first abnormal imaging biomarker in bvFTD. However, the biomarker orderings in bvFTD were predominantly characterised by large uncertainty in the positioning of biomarkers in the disease timeline, with hardly any observable distinction between early and late biomarkers. Online supplemental figure B.3 presents the non-imaging biomarker cascade for the two phenotypes, showing that the uncertainty in the mean cascade in bvFTD is more than in nfvPPA.

## Discussion

In this study, we estimated the cascade of biomarker changes in FTD-*GRN.* We validated our model by delineating the symptomatic mutation carriers from the presymptomatic mutation carriers using the estimated disease severity. We demonstrated that language and NfL levels are the earliest biomarkers to become abnormal in the FTD-*GRN* spectrum. Other early biomarkers were the white matter microstructure of the thalamic radiation and the cognitive domain of attention and mental processing speed.

Our findings support other studies that proposed NfL as an early biomarker for disease onset in FTD-*GRN*.^9 10^ We demonstrated that the left anterior thalamic radiation also degenerated early. This is also supported by previous studies which suggested that white matter microstructure markers may correlate with changes in NfL.^9 28^ Cognitive changes in attention, mental processing speed and executive functioning occurred relatively early in the estimated disease progression timeline. This corresponds well with the early white matter changes (ie, NfL and fractional anisotropy changes), as attention and processing speed are cognitive functions that highly depend on the integrity of axons and their myelin sheaths.^29 30^ The early involvement of these biomarkers point towards axonal degeneration as one of the first pathological processes in *GRN* mutation carriers. However, it must be noted that the estimated cascade shows the sequence of biomarker events when they are detectably abnormal. One of the important factors that affects the detectability of biomarker abnormality in a cross-sectional dataset is the overlap between the normal and abnormal biomarker distributions. Therefore, the presented cross-sectional model cannot provide insight into the sequence of earliest (hardly detectable) changes in the carriers’ biomarker levels. Figure 2 showed that the overlap in cognitive biomarkers was relatively smaller than the overlap in neuroimaging biomarkers, which could explain the relative early positioning of the cognitive biomarker events.

With the differential phenotypic analysis, we estimated the biomarker cascade for nfvPPA and bvFTD patients. Strikingly, language functions deteriorated early in both nfvPPA and bvFTD. While not currently embedded in the clinical criteria for bvFTD,^16^ our results demonstrate the importance of decreased language functions in both phenotypes. This is in line with multiple previous studies.^31–33^ In addition, multiple determinants of the complex language network were also affected early, for example, the left insula and uncinate fasciculus.^34^ While language deficits were estimated as the first detectable abnormal biomarker, the overlap with the second, the elevation in NfL levels, complicates distinguishing the timeline of these disease events. Furthermore, as depicted in figure 2, (subtle) language deficits were less specific for disease onset than NfL levels. However, the high sensitivity of the language biomarker in our study and the relative uncomplicated administration of language tests (compared with neuroimaging techniques, for example) offer potential for longitudinal research in the preclinical stage of FTD-*GRN*—ideally in combination with NfL levels.

For nfvPPA, NfL levels and other cognitive domains became abnormal in early disease stages, consistent with findings from previous studies.^9 10 35^ In addition, we showed that left hemispheric tracts and regions were affected in nfvPPA patients before right regions, accordant with the previously reported strong involvement of the left hemisphere in primary progressive aphasia.^36 37^ We showed that NfL levels and cognitive domains may be possible biomarkers for disease onset, while neuroimaging markers were highly correlated with clinical indicators of progression (years since onset, FTD-CDR-SOB).

For bvFTD, however, the biomarker cascade was characterised by large uncertainty, and the estimated disease severities did not correlate with actual years since onset or FTD-CDR-SOB. This uncertainty could indicate large neuroanatomical heterogeneities between bvFTD patients. Differences in neuroanatomical atrophy patterns have been associated with FTD-*GRN* patients before.^5 6^ Here, we demonstrated that this anatomical heterogeneity is predominantly associated with the bvFTD phenotype, while nfvPPA patients showed a clear pattern of left hemispheric degeneration before the right hemisphere was affected. Furthermore, bvFTD patients present with cognitive symptoms such as impaired social conduct and executive function but can also have severe memory problems. In summary, within the group of bvFTD, spatial and temporal brain degeneration and cognitive changes are more heterogeneous than in the nfvPPA group.

From a methodological point of view, the strength of this paper lies in the introduction of the Siamese GMM approach in DEBM. We showed that Siamese GMM reduces the positional variance in neuroimaging biomarkers, most notably in the right insula, the right anterior thalamic radiation and the right superior longitudinal fasciculus. This is because GMM is known to be unstable in the presence of biomarkers with a large overlap between the normal and abnormal Gaussians.^13^ This is often the case in biomarkers becoming abnormal late in the disease and having very few samples representative of the typical abnormal values expected in the disease. The joint GMM in the Siamese counterpart exploits the knowledge that FTD-*GRN* is generally an asymmetric brain disease, and uses the neuroimaging biomarkers that become abnormal early in the disease process to aid the GMM of its hemispheric counterpart that becomes abnormal far later in the disease process. Another strong point about the DEBM model is that it infers disease progression from cross-sectional data, which is more readily available than longitudinal data, especially in a rare disease as FTD-*GRN*.

From the clinical point of view, a major strength of our study is the large, well-defined cohort of presymptomatic and symptomatic *GRN* mutation carriers, and availability of multimodal (ie, fluid, imaging and cognitive) biomarkers. Although we did not have fluid-attenuated inversion recovery (FLAIR) or T2 imaging data available for the current study, it would be interesting to incorporate white matter lesions in a future version of the model, as a number of studies have indicated the presence of white matter lesions in FTD-*GRN* carriers.^38^ Additionally, including functional neuroimaging, measures in future studies possibly provide new insights into the temporal biomarker sequence and underlying disease mechanism as well. Recent papers have addressed functional changes in FTD-*GRN*, showing thalamic-cortical hyperconnectivity in early preclinical stages^39^ and presymptomatic abnormalities in neurophysiology.^40^

A minor limitation in our study is the difference in mean age between the non-carrier, presymptomatic and symptomatic mutation carrier groups. We adjusted for this in the analysis rather than matching the groups. It should be noted that the small sample size may have caused a large part of the uncertainty of our model, especially in the case of missing (neuroimaging) biomarkers. Our bvFTD and nfvPPA samples due to *GRN* mutations were relatively large compared with previous studies.^41^ However, the DEBM model would improve substantially if the phenotypic samples were larger, as we could only include symptomatic subjects for the phenotypic analysis. Uncertainties in the estimation of the phenotypic biomarker cascades may be improved with upcoming longitudinal data, when some of the converted mutation carriers can be included in the phenotypic models.

In conclusion, with this DEBM study in the FTD-*GRN* spectrum, we were able to demonstrate that language functions and NfL levels are the earliest abnormal biomarkers, regardless of phenotype. However, bvFTD show more heterogeneity and uncertainty in disease progression, pointing towards more variability in biomarkers than nfvPPA. Our analyses suggest axonal degeneration and damage to the language network as the earliest biomarkers in *GRN* mutation carriers, which could potentially be used as endpoints in clinical trials for disease modifying treatments. Future efforts should be directed at confirmation and validation of these findings with longitudinal data. Validation of these results in an external cohort such as the Longitudinal Evaluation of Familial Frontotemporal Dementia Subjects (LEFFTDS)^42^ could further aid in confirming these results and elucidate any ethnic variations in the disease progression timeline. We expect that DEBM modelling will benefit individual prediction of symptom onset in the future, and may optimise selection of eligible mutation carriers for clinical trials.

10.1136/jnnp-2020-323541.supp3 Supplementary data

## Data Availability

Data are available upon reasonable request. The raw data of this project are part of Genetic Frontotemporal dementia Initiative and are not publicly available. Data can be accessed upon reasonable request to JCVS (j.c.vanswieten@erasmusmc.nl) and JDR (j.rohrer@ucl.ac.uk). The code for discriminative event-based modelling is available and can be downloaded from https://github.com/88vikram/pyebm/.
