# Supplementary material for: Modelling the cascade of biomarker changes in GRN-related frontotemporal dementia
Source: J Neurol Neurosurg Psychiatry. 2021 Jan 15;92(5):494–501. doi: 10.1136/jnnp-2020-323541 (PMC8053353; doi:10.1136/jnnp-2020-323541)
Supplement: Supplementary data [file jnnp-2020-323541supp001.pdf]

Appendix A. Biomarkers

Biomarker selection

For biomarker selection, we extensively searched for relevant literature about presymptomatic FTD-*GRN* in Pubmed. We reviewed all empirical studies that included at least a presymptomatic *GRN* mutation carrier group. Next, we determined which biomarkers were frequently reported as abnormal in previous empirical studies and included these biomarkers accordingly, restricted to fluid biomarkers, grey matter brain regions, white matter tracts, and cognition. The selected biomarkers were: serum NfL [1-3], MMSE [4-6], cognitive domains of language, attention and processing speed, executive functioning, and social cognition [5, 7-9]; left and right volumes of the insula, frontal lobe, parietal lobe and the temporal lobe [4, 6, 10-16] [17-19] [20, 21]; white matter tracts: left and right fractional anisotropy of anterior thalamic radiation, superior longitudinal fasciculus, uncinate fasciculus, and forceps minor [10, 17-19, 22, 23]. Although the *GRN* mutation affects plasma progranulin protein levels, these levels were not selected as biomarker, as research has shown that these remain stable in both the presymptomatic and symptomatic stage [6, 24].

MRI processing and ROI calculation

An overview of MRI acquisition parameters is presented in Appendix Table 1. The standard voxel-based morphometry pipeline from FSL [25-27] was used to process T1-weighted images. In brief, the brain was extracted from the images, and we carefully checked the brain extraction for missing brain tissue and areas of non-brain tissue, and adjusted the image accordingly. We corrected RF inhomogeneities by bias field correction with a Markov random field model and subsequently segmented the brain in grey matter, white matter, and cerebrospinal fluid images [28]. A study specific grey matter template was created in standard space using a balanced set of subjects, and all grey matter segmentations were registered to this template with non-linear registration,

and then corrected for any local expansion or contraction by modulation of the Jacobian warp field [26]. Last, an isotropic Gaussian kernel with a sigma of 3mm was applied for smoothing of the grey matter images. Total intracranial volume (TIV) was calculated as the sum of the volumes from grey matter, white matter and cerebrospinal fluid in standard space. The structures from the MNI-atlas were used as grey matter ROIs. We extracted volumetric measurements from the ROIs by registering the structural MNI-atlas [29] to the grey matter images in standard space, and multiplying the grey matter density of the ROI with the total volume of the ROI, resulting in the grey matter volume within the ROI. Left and right regions were considered separately.

Diffusion tensor images were corrected for motion artefacts and eddy currents by alignment to the b=0 image, and subsequently, the tensor was fitted at each voxel to create fractional anisotropy (FA) images. The FA images were processed with the tract-based spatial statistics (TBSS) pipeline as implemented in FSL [30]. Using non-linear registration, the images were aligned to the FMRIB58_FA template and then averaged into a mean FA image. The mean FA image was thresholded at 0.2 and thinned into a white matter skeleton. All individual FA images were projected onto this skeleton, resulting in skeletonized FA data for each participant. The probabilistic tracts from the Johns Hopkins University atlas [31] were applied as white matter ROIs to the skeleton mask, and the masked ROIs were used to extract FA values from the individual tracts. Left and right tracts were considered separately.

Table A.1. MRI acquisition protocols

	**Rotterdam 1**	**Rotterdam 2**	**Brescia**	**Barcelona**
**N** (s/p/nc)	3/22/24	5/9/6	7/17/0	1/6/1
**Scanner**	Philips Achieva 3T	Philips Achieva 3T	Siemens Skyra	Siemens Trio Tim
**Head Coil**	8 channel SENSE	32 channel SENSE	32 channel	64 channel
T1 weighted imaging				
**TR**	9.8 ms	6.8 ms	2000 ms	2000 ms
**TE**	4.6 ms	3.1 ms	2.9 ms	2.9 ms
**FOV**	224x168 mm	256x256 mm	282x282 mm	282x282 mm
**Voxel size**	0.88x0.88x1.2 mm	1.1mm^3	1.1 mm^3	1.1mm^3
**Flip angle**	8°	8°	8°	8°
**Slices**	140	207	208	208
Diffusion tensor imaging				
**TR**	8250 ms	7000 ms	7300 ms	7300 ms
**TE**	80 ms	69 ms	90 ms	90 ms
**FOV**	256x256 mm	240x240 mm	240x240 mm	240x240 mm
**Voxel size**	2x2x2mm	2.5x2.5x2.5mm	2.5x2.5x2.5mm	2.5x2.5x2.5mm
**Slices**	70	59	59	59
**Directions**	60	68	68	68
**B-values**	0/1000 s/mm^2	0/1000 s/mm^2	0/1000 s/mm^2	0/1000 s/mm^2

Numbers are subjects included after quality check. Abbreviations: s = symptomatic, p = presymptomatic, nc = non-carrier, TR = repetition time, TE = echo time, FOV = field of view.

Cognitive assessment

The following cognitive tests were performed, depending on the protocol from the local site. For language, the Boston Naming Task [32] and semantic fluency (animals) [33] were used. Tests concerning attention and processing speed were the Trail making test part A [34], Stroop part 1 and 2 [35], symbol substitution [36], letter digit substitution task [37], and forward digit span [36]. For executive functioning, we used Trail making test part B [34], Stroop task part 3 [35], phonological fluency [33] and digit span backwards [36]. Tests for social cognition were the Ekman faces test [38], emotion recognition from the mini social cognition and emotional assessment (MINI-SEA) [39], and Happé cartoon task [40]. Raw scores from tests in which a higher score indicates worse performance were reversed (i.e. Trail making test, Stroop). We transformed all raw test scores to z-scores, based on the mean and standard deviation of the non-carriers. Subsequently, cognitive domains were composed as the mean z-score of all available tests within that domain per individual, disregarding missing tests.

Table A.2. Availability and characteristics of cognitive data

	Symptomatic						Presymptomatic	
	Total (n=35)[*]		bvFTD (n=17)		nfvPPA (n=16)		N=56	
	N	*Mean ± SD*	*N*	*Mean ± SD*	*N*	*Mean ± SD*	*N*	*Mean ± SD*
**MMSE**	29	-3.07 ± 1.50	15	-3.26 ± 1.69	14	-2.87 ± 1.28	55	0.22 ± 1.01
**Language**	32	-2.86 ± 1.37	16	-2.69 ± 1.58	14	-3.00 ± 1.24	55	0.28 ± 1.09
Boston naming test	25	-1.97 ± 1.32	13	-1.75 ± 1.44	12	-2.21 ± 1.19	55	0.57 ± 1.37
Semantic fluency	31	-3.28 ± 1.38	15	-3.14 ± 1.57	14	-3.45 ± 1.30	55	0.00 ± 1.31
**Attention, concentration and mental processing speed**	33	-2.35 ± 1.17	16	-2.43 ± 1.32	15	-2.26 ± 1.12	55	-0.05 ± 0.75
TMT-A	32	-2.65 ± 1.62	16	-2.91 ± 1.63	14	-2.23 ± 1.66	55	-0.01 ± 0.92
Stroop card 1&2	17	-3.05 ± 2.18	10	-2.96 ± 2.20	7	-3.18 ± 2.32	55	0.14 ± 1.02
LDST	4	-2.06 ± 1.59	2	-2.09 ± 1.99	2	-2.03 ± 1.90	17	0.22 ± 0.70
Symbol substitution	17	-2.35 ± 1.41	7	-3.01 ± 1.13	10	-1.89 ± 1.45	22	0.00 ± 1.29
Digit span forward	31	-1.66 ± 1.05	15	-1.41 ± 1.29	14	-1.95 ± 0.74	55	-0.26 ± 0.95
**Executive functioning**	32	-2.33 ± 0.97	15	-2.23 ± 1.19	15	-2.37 ± 0.79	55	-0.03 ± 0.75
TMT-B	28	-2.63 ± 0.97	13	-2.50 ± 1.11	13	-2.69 ± 0.89	55	0.03 ± 0.79

Stroop card 3	14	-3.84 ± 2.29	8	-3.73 ± 2.50	6	-3.98 ± 2.20	55	-0.36 ± 1.05
Phonological fluency	29	-2.12 ± 0.95	14	-1.92 ± 1.02	15	-2.30 ± 0.87	55	0.30 ± 1.36
Digit span backwards	30	-1.65 ± 1.14	15	-1.61 ± 1.44	13	-1.61 ± 0.75	55	-0.08 ± 1.11
**Social cognition**	15	-1.87 ± 0.76	7	-2.15 ± 0.92	8	-1.62 ± 0.52	51	-0.10 ± 1.02
Ekman faces	3	-0.70 ± 0.60	2	-0.37 ± 0.18	1	-1.36 ± N/A	26	0.14 ± 0.89
Mini-SEA Emotion Recognition	10	-1.98 ± 0.83	3	-2.65 ± 1.13	7	-1.69 ± 0.52	22	-0.62 ± 0.98
HappeTOM	5	-2.07 ± 0.86	4	-2.32 ± 0.75	1	-1.05 ± N/A	28	0.42 ± 0.77
Happe non TOM	5	-1.65 ± 0.81	4	-1.81 ± 0.84	1	-1.03 ± N/A	28	0.34 ± 1.20

Abbreviations: bvFTD = behavioural variant frontotemporal dementia, nfvPPA = non-fluent variant primary progressive aphasia, MMSE = mini mental state examination, TMT = trail making test, LDST = letter digit substitution task, mini-SEA = mini social cognition and emotional assessment, TOM = theory of mind. Values are mean z-scores ± standard deviation based on non-carriers, uncorrected for confounding factors. * The two remaining participants presented with cortico-basal degeneration.

Biomarker statistics

Before modelling, we checked skewed distributions in the biomarkers with the following graphs and tests: histograms, q-q plots, skewness and kurtosis values (values between 2 and –2 indicate normality), Kolmogorov-Smirnov and Shapiro-Wilk's tests (values above 0.05 indicate normality). When three or more tests indicated skewness, the distributions were adjusted using log-transformations (log10), i.e. neurofilament light chain levels, MMSE, BNT, Trail Making Test, Stroop, facial emotion recognition. In the case of cognitive tests, log-transformation was performed before transforming raw scores to z-scores.

Biomarker characteristics and statistical differences between groups are presented in Table A.3. Symptomatic mutation carriers had higher NfL levels, lower grey matter volumes, impaired white matter microstructure, and worse cognitive functions than both presymptomatic mutation carriers and non-carriers in all selected biomarkers. Post-hoc analysis revealed that these differences in biomarkers were specifically driven by the bvFTD patients. For nfvPPA patients, we found higher NfL levels and worse cognitive performance than both presymptomatic mutation carriers and non-carriers. NfvPPA patients showed smaller grey matter volumes than both presymptomatic mutation carriers and non-carriers, especially in left-sided ROIs, and lower fractional anisotropy levels in the left anterior thalamic radiation, left uncinate fasciculus, and the forceps minor. The volume of the right frontal lobe was smaller in nfvPPA patients compared with presymptomatic mutation carriers. Furthermore, bvFTD patients had smaller volumes of the right frontal and temporal lobe than nfvPPA patients, and lower fractional anisotropy values in the forceps minor, left superior longitudinal fasciculus and right uncinate fasciculus. There were no differences in any of the selected biomarkers between presymptomatic mutation carriers and non-carriers.

Table A.3. Biomarker characteristics after correction for confounding factors

		Symptomatic			Presymptomatic
		Total	bvFTD	nfvPPA	
Neurofilament light chain		1.90 ± 0.25*	1.89 ± 0.23*	1.91 ± 0.28†	1.10 ± 0.22
GM volume	*Left frontal lobe*	-2.75 ± 1.8*	-3.42 ± 2.06*	-2.46 ± 1.40†	0.30 ± 0.65
	Right frontal lobe	-1.72 ± 1.79*	-2.76 ± 1.43*,‡	-0.93 ± 1.79§	0.30 ± 0.65
	Left insula	-2.32 ± 1.56*	-2.45 ± 1.79*	-2.35 ± 1.51†	-0.32 ± 0.95
	Right insula	-1.02 ± 1.13*	-1.47 ± 1.26*	-0.74 ± 0.98	-0.08 ± 0.84
	Left parietal lobe	-1.87 ± 1.11*	-2.18 ± 1.39*	-1.74 ± 0.84†	-0.03 ± 1.02
	Right parietal lobe	-1.19 ± 2.00*	-1.42 ± 2.08*	-0.89 ± 2.15	-0.06 ± 0.96
	Left temporal lobe	-2.97 ± 2.42*	-3.21 ± 2.59*	-2.98 ± 2.51†	-0.19 ± 0.96
	Right temporal lobe	-1.14 ± 2.66*	-2.22 ± 3.40*,‡	-0.12 ± 1.69	-0.08 ± 0.94
FA	*Left anterior thalmic radiation*	-2.28 ± 1.34*	-2.73 ± 1.60*	-1.77 ± 0.98†	-0.33 ± 0.95
	Right anterior thalamic radiation	-1.24 ± 1.23*	-1.78 ± 1.51*	-0.66 ± 0.66	-0.27 ± 0.77
	Forceps Minor	-3.00 ± 1.52*	-4.01 ± 1.52*,‡	-2.08 ± 0.96†	0.46 ± 0.93
	Left superior longitudinal fasciculus	-1.50 ± 1.39*	-2.42 ± 1.28*,‡	-0.61 ± 0.96	0.02 ± 0.88
	Right superior longitudinal fasciculus	-1.14 ± 1.12*	-1.47 ± 1.14*	-0.74 ± 1.06	-0.11 ± 0.60
	Left uncinated fasciculus	-2.63 ± 1.15*	-3.00 ± 1.43*	-2.29 ± 0.88†	-0.35 ± 0.86
	Right uncinate fasciculus	-1.92 ± 2.16*	-3.19 ± 2.07*,‡	-0.77 ± 1.74	-0.51 ± 1.12
MMSE		-2.71 ± 1.19*	-2.71 ± 1.28*	-2.71 ± 1.14†	0.06 ± 0.91
Attention and processing speed		-2.06 ± 1.09*	-2.11 ± 1.15*	-2.05 ± 1.12†	-0.22 ± 0.65

Executive functioning	-2.12 ± 0.88*	-2.00 ± 0.99*	-2.24 ± 0.82†	-0.14 ± 0.72
Language	-2.54 ± 1.23*	-2.35 ± 1.33*	-2.84 ± 1.17†	0.13 ± 0.97
Social cognition	-1.89 ± 0.64*	-2.13 ± 0.74*	-1.52 ± 0.42†	-0.19 ± 0.96

Abbreviations: bvFTD = behavioural variant frontotemporal dementia, nfvPPA = non-fluent variant primary progressive aphasia, GM volume = grey matter volume, FA = fractional anisotropy, MMSE = Mini Mental State Examination. Values are mean z-score (based on non-carriers) ± standard deviation, after correction for confounding factors of age, gender, MRI protocol, and years of education.

* Both the entire group of symptomatic mutation carriers and only bvFTD patients significantly differed from presymptomatic mutation carriers as well as non-carriers (*p<0.05*, Bonferroni corrected)

† Significant difference between nfvPPA patients and presymptomatic mutation carriers as well as non-carriers (*p<0.05*, Bonferroni corrected)

‡ Significant difference between bvFTD patients and nfvPPA patients (*p<0.05*, Bonferroni corrected)

¶ Significant difference between nfvPPA patients and presymptomatic mutation carriers (*p<0.05*, Bonferroni corrected)

References

1. Meeter, L.H., et al., *Neurofilament light chain: a biomarker for genetic frontotemporal dementia.* Ann Clin Transl Neurol, 2016. **3**(8): p. 623-36.
2. Rohrer, J.D., et al., *Serum neurofilament light chain protein is a measure of disease intensity in frontotemporal dementia.* Neurology, 2016. **87**(13): p. 1329-36.
3. van der Ende, E.L., et al., *Serum neurofilament light chain in genetic frontotemporal dementia: a longitudinal, multicentre cohort study.* Lancet Neurol, 2019. **18**(12): p. 1103-1111.
4. Rohrer, J.D., et al., *Presymptomatic cognitive and neuroanatomical changes in genetic frontotemporal dementia in the Genetic Frontotemporal dementia Initiative (GENFI) study: a cross-sectional analysis.* Lancet Neurol, 2015. **14**(3): p. 253-62.
5. Jiskoot, L.C., et al., *Longitudinal cognitive biomarkers predicting symptom onset in presymptomatic frontotemporal dementia.* J Neurol, 2018.
6. Benussi, A., et al., *Clinical and biomarker changes in presymptomatic genetic frontotemporal dementia.* Neurobiol Aging, 2019. **76**: p. 133-140.
7. Barandiaran, M., et al., *Neuropsychological features of asymptomatic c.709-1G>A progranulin mutation carriers.* J Int Neuropsychol Soc, 2012. **18**(6): p. 1086-90.
8. Barandiaran, M., et al., *Longitudinal Neuropsychological Study of Presymptomatic c.709-1G>A Progranulin Mutation Carriers.* J Int Neuropsychol Soc, 2019. **25**(1): p. 39-47.
9. Jiskoot, L.C., et al., *Presymptomatic cognitive decline in familial frontotemporal dementia: A longitudinal study.* Neurology, 2016. **87**(4): p. 384-91.
10. Borroni, B., et al., *Brain magnetic resonance imaging structural changes in a pedigree of asymptomatic progranulin mutation carriers.* Rejuvenation Res, 2008. **11**(3): p. 585-95.
11. Cash, D.M., et al., *Patterns of gray matter atrophy in genetic frontotemporal dementia: results from the GENFI study.* Neurobiol Aging, 2017. **62**: p. 191-196.

12. Premi, E., et al., *Subcortical and Deep Cortical Atrophy in Frontotemporal Dementia due to Granulin Mutations.* Dement Geriatr Cogn Dis Extra, 2014. **4**(1): p. 95-102.

13. Young, A.L., et al., *Uncovering the heterogeneity and temporal complexity of neurodegenerative diseases with Subtype and Stage Inference.* Nat Commun, 2018. **9**(1): p. 4273.

14. Jacova, C., et al., *Anterior brain glucose hypometabolism predates dementia in progranulin mutation carriers.* Neurology, 2013. **81**(15): p. 1322-31.

15. Popuri, K., et al., *Gray matter changes in asymptomatic C9orf72 and GRN mutation carriers.* Neuroimage Clin, 2018. **18**: p. 591-598.

16. Moreno, F., et al., *Distinctive age-related temporal cortical thinning in asymptomatic granulin gene mutation carriers.* Neurobiol Aging, 2013. **34**(5): p. 1462-8.

17. Dopper, E.G., et al., *Structural and functional brain connectivity in presymptomatic familial frontotemporal dementia.* Neurology, 2014. **83**(2): p. e19-26.

18. Jiskoot, L.C., et al., *Longitudinal multimodal MRI as prognostic and diagnostic biomarker in presymptomatic familial frontotemporal dementia.* Brain, 2019. **142**(1): p. 193-208.

19. Panman, J.L., et al., *Gray and white matter changes in presymptomatic genetic frontotemporal dementia: a longitudinal MRI study.* Neurobiol Aging, 2019. **76**: p. 115-124.

20. Pievani, M., et al., *Pattern of structural and functional brain abnormalities in asymptomatic granulin mutation carriers.* Alzheimers Dement, 2014. **10**(5 Suppl): p. S354-S363 e1.

21. Caroppo, P., et al., *Lateral Temporal Lobe: An Early Imaging Marker of the Presymptomatic GRN Disease?* J Alzheimers Dis, 2015. **47**(3): p. 751-9.

22. Jiskoot, L.C., et al., *Presymptomatic white matter integrity loss in familial frontotemporal dementia in the GENFI cohort: A cross-sectional diffusion tensor imaging study.* Ann Clin Transl Neurol, 2018. **5**(9): p. 1025-1036.

23. Olm, C.A., et al., *Longitudinal structural gray matter and white matter MRI changes in presymptomatic progranulin mutation carriers.* Neuroimage Clin, 2018. **19**: p. 497-506.
24. Meeter, L.H., et al., *Progranulin Levels in Plasma and Cerebrospinal Fluid in Granulin Mutation Carriers.* Dement Geriatr Cogn Dis Extra, 2016. **6**(2): p. 330-340.
25. Douaud, G., et al., *Anatomically related grey and white matter abnormalities in adolescent-onset schizophrenia.* Brain, 2007. **130**(Pt 9): p. 2375-86.
26. Jenkinson, M., et al., *Fsl.* Neuroimage, 2012. **62**(2): p. 782-90.
27. Smith, S.M., et al., *Advances in functional and structural MR image analysis and implementation as FSL.* Neuroimage, 2004. **23 Suppl 1**: p. S208-19.
28. Zhang, Y., M. Brady, and S. Smith, *Segmentation of brain MR images through a hidden Markov random field model and the expectation-maximization algorithm.* IEEE Trans Med Imaging, 2001. **20**(1): p. 45-57.
29. Collins, D.L., Holmes, C.J., Peters, T.M., Evans, A.C., *Automated 3-D model-based neuroanatomical segmentation.* Human Brain Mapping, 1995. **3**(3): p. 190-208.
30. Smith, S.M., et al., *Tract-based spatial statistics: voxelwise analysis of multi-subject diffusion data.* Neuroimage, 2006. **31**(4): p. 1487-505.
31. Mori, S., Wakana, S, van Zijl, P.C.M, Nagae-Poetscher, L.M., *MRI atlas of Human White Matter*. 2005, Amsterdam, the Netherlands: Elsevier.
32. Kaplan, E., Goodglass, H., Weintraub, S., *Boston Naming Test*. 1983, Philadelphia: Lea & Febiger.
33. Thurstone, L.L., Thurstone, T.G., *Primary Mental Abilities*. 1962, Chicago: University of Chicago Press.
34. battery, A.i.t., *Manual of directions and scoring*. 1944, Washington, DC: War Department, Adjunct General's Office.
35. Stroop, J., *Studies of interference in serial verbal reaction.* Journal of Experimental Psychology, 1935. **18**: p. 643-662.

36. Wechsler, D., *WAIS-III Technische Handleiding*. 2005, Amsterdam: Harcourt Test Publishers.

37. Jolles, J., Houx, P.K., van Boxtel, M.P.J., Ponds R.W.H.M., *Maastricht aging study: Determinants of cognitive aging*. 1995, Maastricht: Neuropsych Publishers.

38. Ekman, P., Friesen, W.V., *Pictures of facial affect* 1976, Palo Alto, CA: Consulting Psychological Press.

39. Funkiewiez, A., et al., *The SEA (Social cognition and Emotional Assessment): a clinical neuropsychological tool for early diagnosis of frontal variant of frontotemporal lobar degeneration.* Neuropsychology, 2012. **26**(1): p. 81-90.

40. Happe, F., H. Brownell, and E. Winner, *Acquired 'theory of mind' impairments following stroke.* Cognition, 1999. **70**(3): p. 211-40.
