# Supplementary material for: Modelling the cascade of biomarker changes in GRN-related frontotemporal dementia
Source: J Neurol Neurosurg Psychiatry. 2021 Jan 15;92(5):494–501. doi: 10.1136/jnnp-2020-323541 (PMC8053353; doi:10.1136/jnnp-2020-323541)
Supplement: Supplementary data [file jnnp-2020-323541supp002.pdf]

APPENDIX B – DISCRIMINATIVE EVENT BASED MODELLING

DEBM: Gaussian mixture modelling

DEBM uses Gaussian mixture modelling to transform biomarker values to posterior probabilities of them being abnormal. This is done by assuming the probability density functions of normal and abnormal values are represented by Gaussians $N(\mu_{\sim E}, \sigma_{\sim E})$and $N(\mu_E, \sigma_E)$ respectively, where the occurrence of the biomarker abnormality event is denoted by E and the absence of such an event is denoted by $\sim E$.

Gaussian mixture modelling is an optimisation task to estimate these normal and abnormal Gaussians as well as the mixing parameter based on maximum log-likelihood, where the log-likelihood for biomarker B is computed as the summation over all *GRN* mutation carriers in the dataset as follows:

$$L_B = \sum_{\forall j \in Carriers} \log f(B_j)$$

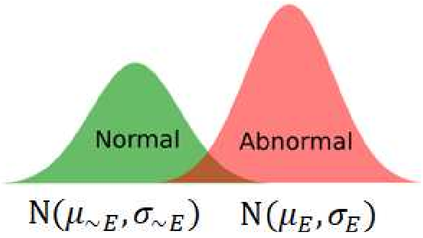

Figure B.1: Illustrations of the Gaussian probability density functions for normal and abnormal values of biomarker B

Here, the likelihood $f(B)$ is computed as follows:

$$f(B) = \theta_{\sim E} p(B|\mu_{\sim E}, \sigma_{\sim E}) + \theta_E p(B|\mu_E, \sigma_E),$$

Where $\theta_{\sim E} + \theta_E = 1$, and the mixing parameters $\theta_{\sim E}$ and θ_E show the relative proportions of the two Gaussians in the dataset. The abnormal Gaussian is initialized using the mean and standard deviation of the symptomatic subjects, while the normal Gaussian is initialized using the non-carriers. Since non-carriers are healthy controls, we fix $\mu_{\sim E}$ and $\sigma_{\sim E}$ to their initialized values and only optimize the remaining parameters in the Gaussian mixture model. The mixing parameter and the Gaussian parameters are optimized alternately until convergence as detailed previously,[1].

For imaging-biomarkers with left and right counter parts, we propose a novel modification to the Gaussian mixture model optimization called Siamese Gaussian mixture model (Siamese GMM). We propose to jointly optimize the parameters of these biomarkers, by taking advantage of symmetry in the brain. The log-likelihood for the joint optimization for the imaging biomarkers I^L and $I^R$is given below:

$$L_I = \sum_{\forall j \in Carriers} \log f\left({I^L}_j\right) + \log f\left({I^R}_j\right)$$

where $f\left({I^L}_j\right)$ and $f\left({I^R}_j\right)$ are expressed mathematically as:

$$f\left({I^L}_j\right) = \theta^L_{\sim E} p\left({I^L}_j \middle| \mu_{\sim E}, \sigma_{\sim E}\right) + \theta^L_E p\left({I^L}_j \middle| \mu_E, \sigma_E\right)$$

$$f\left({I^R}_j\right) = \theta^R_{\sim E} p\left({I^R}_j \middle| \mu_{\sim E}, \sigma_{\sim E}\right) + \theta^R_E p\left({I^R}_j \middle| \mu_E, \sigma_E\right)$$

$\theta^L_{\sim E} + \theta^L_E = 1$ and $\theta^R_{\sim E} + \theta^R_E = 1$. The mixing parameters $(\theta^L_{\sim E}, \theta^L_E, \theta^R_{\sim E}, \theta^R_E)$ and the abnormal Gaussian parameters $(\mu_E,\ \sigma_E)$ are again optimized alternately until convergence,[1]. This joint optimization of the left and right counter parts by sharing the normal and abnormal Gaussians reduces the number of parameters to be optimized, and thus improves the robustness. In case of asymmetrical atrophy patterns, where one of the biomarkers is stronger than the other, the joint optimization also helps in making the GMM more stable for the weaker biomarker.

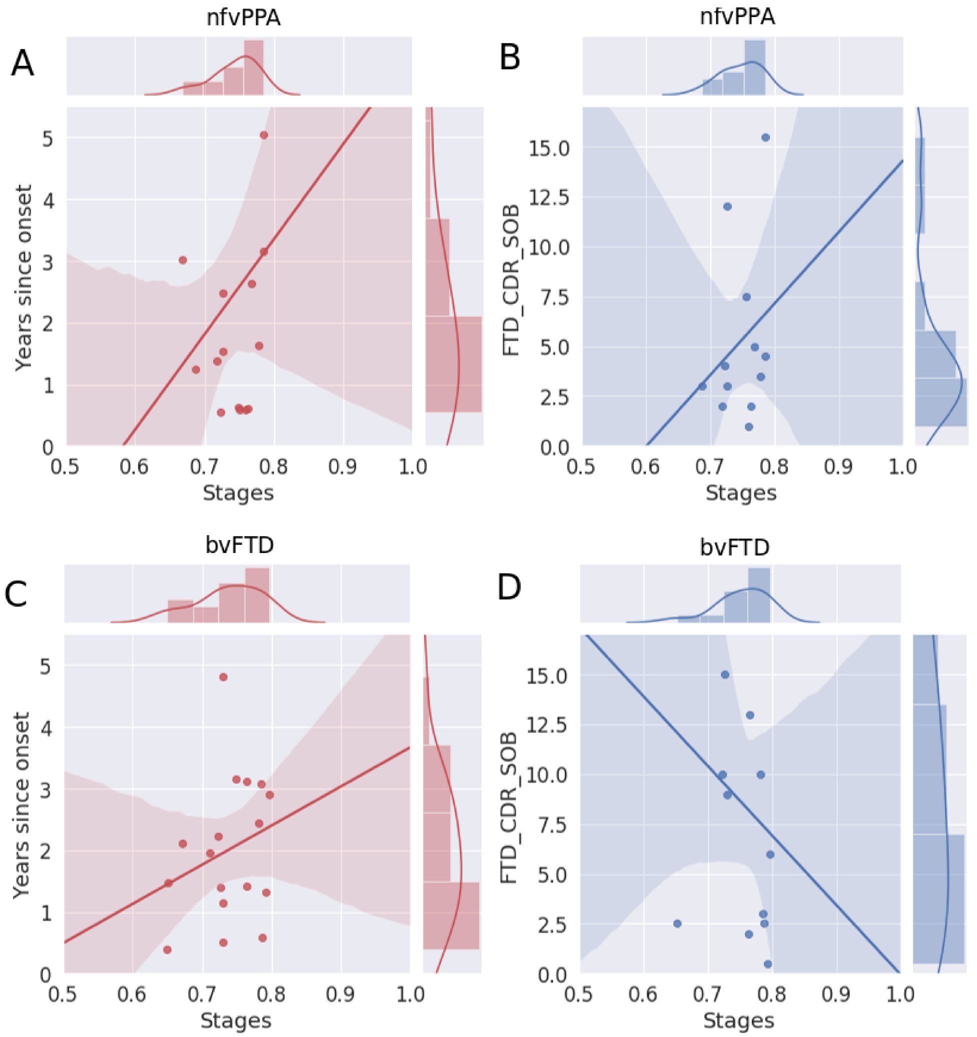

Figure B.2. Correlation of disease severity (as estimated by non-imaging DEBM using cross-validation) with years since onset and FTD-CDR-SOB. The 2D scatter plots in figures A and C show the correlations of disease severity with years since onset, for symptomatic nfvPPA and bvFTD subjects respectively. The 2D scatter plot in figures B and D show the correlations of disease severity with FTD-CDR-SOB. The plot on top of each subfigure shows the probability density function of the disease stages. The plots on the right of figures A and C show the probability density functions of years since symptom onset. The plots on the right of figures B and D show the probability density function of FTD-CDR-SOB.

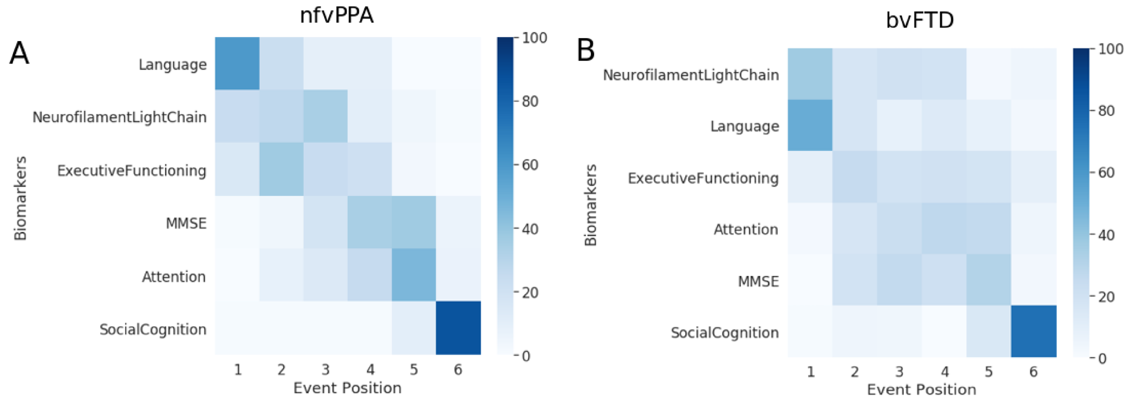

Figure B.3. Cascade of non-imaging biomarker changes in nfvPPA (A) and bvFTD (B) subjects along with the uncertainty associated with it. The biomarkers are ordered based on the position in the estimated cascade. The color-map is based on the number of times a biomarker is at a position in 100 repetitions of bootstrapping.

References

1. Venkatraghavan, V., Bron, E.E., Wiessen, W.J., et al., *Disease progression timeline estimation for Alzheimer's disease using discriminative event based modeling.* Neuroimage, 2019. **186**: p. 518-532.
