# Supplementary material for: Modelling the cascade of biomarker changes in GRN-related frontotemporal dementia
Source: J Neurol Neurosurg Psychiatry. 2021 Jan 15;92(5):494–501. doi: 10.1136/jnnp-2020-323541 (PMC8053353; doi:10.1136/jnnp-2020-323541)
Supplement: Supplementary data [file jnnp-2020-323541supp003.pdf]

Appendix C – GENFI consortium members

**Author**	**Affiliation**
Sónia Afonso	Instituto Ciencias Nucleares Aplicadas a Saude, Universidade de Coimbra, Coimbra, Portugal
Maria Rosario Almeida	Faculty of Medicine, University of Coimbra, Coimbra, Portugal
Sarah Anderl-Straub	Department of Neurology, University of Ulm, Ulm, Germany
Christin Andersson	Department of Clinical Neuroscience, Karolinska Institutet, Stockholm, Sweden
Anna Antonell	Alzheimer’s disease and Other Cognitive Disorders Unit, Neurology Service, Hospital Clínic, Barcelona, Spain
Silvana Archetti	Biotechnology Laboratory, Department of Diagnostics, Spedali Civili Hospital, Brescia, Italy
Andrea Arighi	Fondazione IRCCS Ca’ Granda Ospedale Maggiore Policlinico, Neurodegenerative Diseases Unit, Milan, Italy; University of Milan, Centro Dino Ferrari, Milan, Italy
Mircea Balasa	Alzheimer’s disease and Other Cognitive Disorders Unit, Neurology Service, Hospital Clínic, Barcelona, Spain
Myriam Barandiaran	Cognitive Disorders Unit, Department of Neurology, Donostia University Hospital, San Sebastian, Gipuzkoa, Spain; Neuroscience Area, Biodonostia Health Research Insitute, San Sebastian, Gipuzkoa, Spain
Nuria Bargalló	Imaging Diagnostic Center, Hospital Clínic, Barcelona, Spain
Robart Bartha	Department of Medical Biophysics, The University of Western Ontario, London, Ontario, Canada; Centre for Functional and Metabolic Mapping, Robarts Research Institute, The University of Western Ontario, London, Ontario, Canada
Benjamin Bender	Department of Diagnostic and Interventional Neuroradiology, University of Tübingen, Tübingen, Germany
Sandra Black	Sunnybrook Health Sciences Centre, Sunnybrook Research Institute, University of Toronto, Toronto, Canada
Chris Butler	Department of Clinical Neurology, University of Oxford, Oxford, UK
Martina Bocchetta	Dementia Research Centre, Department of Neurodegenerative Disease, UCL Institute of Neurology, Queen Square, London, UK
Sergi Borrego-Ecija	Alzheimer’s disease and Other Cognitive Disorders Unit, Neurology Service, Hospital Clínic, Barcelona, Spain
Jose Bras	Dementia Research Institute, Department of Neurodegenerative Disease, UCL Institute of Neurology, Queen Square, London, UK
Rose Bruffaerts	Laboratory for Cognitive Neurology, Department of Neurosciences, KU Leuven, Leuven, Belgium
Paola Caroppo	Fondazione IRCCS Istituto Neurologico Carlo Besta, Milano, Italy
David Cash	Dementia Research Centre, Department of Neurodegenerative Disease, UCL Institute of Neurology, Queen Square, London, UK
Miguel Castelo-Branco	Faculty of Medicine, University of Coimbra, Coimbra, Portugal

Rhian Convery	Dementia Research Centre, Department of Neurodegenerative Disease, UCL Institute of Neurology, Queen Square, London, UK
Thomas Cope	Department of Clinical Neuriscience, University of Cambridge, Cambridge, UK
Adrian Danek	Neurologische Klinik und Poliklinik, Ludwig-Maximilians-Universität, Munich, German Center for Neurodegenerative Diseases (DZNE), Munich, Germany
María de Arriba	Neuroscience Area, Biodonostia Health Research Insitute, San Sebastian, Gipuzkoa, Spain
Alexandre de Mendonça	Faculty of Medicine, University of Lisbon, Lisbon, Portugal
Giuseppe Di Fede	Fondazione IRCCS Istituto Neurologico Carlo Besta, Milano, Italy
Zigor Díaz	CITA Alzheimer, San Sebastian, Gipuzkoa, Spain
Simon Ducharme	Department of Psychiatry, McGill University Health Centre, McGill University, Montreal, Québec, Canada
Diana Duro	Faculty of Medicine, University of Coimbra, Coimbra, Portugal
Chiara Fenoglio	Fondazione IRCCS Ca' Granda Ospedale Maggiore Policlinico, Neurodegenerative Diseases Unit, Milan, Italy; University of Milan, Centro Dino Ferrari, Milan, Italy
Catarina B. Ferreira	Laboratory of Neurosciences, Institute of Molecular Medicine, Faculty of Medicine, University of Lisbon, Lisbon, Portugal
Elizabeth Finger	Department of Clinical Neurological Sciences, University of Western Ontario, London, ON, Canada
Toby Flanagan	Faculty of Biology, Medicine and Health, Division of Neuroscience and Experimental Psychology, University of Manchester, Manchester, UK
Nick Fox	Dementia Research Centre, Department of Neurodegenerative Disease, UCL Institute of Neurology, Queen Square, London, UK
Morris Freedman	Baycrest Health Sciences, Rotman Research Institute, University of Toronto, Toronto, Canada
Giorgio Fumagalli	Fondazione IRCCS Ca' Granda Ospedale Maggiore Policlinico, Neurodegenerative Diseases Unit, Milan, Italy; University of Milan, Centro Dino Ferrari, Milan, Italy; Department of Neurosciences, Psychology, Drug Research and Child Health (NEUROFARBA), University of Florence, Florence, Italy
Alazne Gabilondo	Neuroscience Area, Biodonostia Health Research Insitute, San Sebastian, Gipuzkoa, Spain
Daniela Galimberti	Fondazione IRCCS Ca' Granda Ospedale Maggiore Policlinico, Neurodegenerative Diseases Unit, Milan, Italy
Roberto Gasparotti	Neuroradiology Unit, University of Brescia, Brescia, Italy
Serge Gauthier	Alzheimer Disease Research Unit, McGill Centre for Studies in Aging, Department of Neurology & Neurosurgery, McGill University, Montreal, Québec, Canada
Stefano Gazzina	Centre for Neurodegenerative Disorders, Neurology Unit, Department of Clinical and Experimental Sciences, University of Brescia, Brescia, Italy
Alexander Gerhard	Institute of Brain, Behaviour and Mental Health, The University of Manchester, Withington, Manchester, UK
Giorgio Giaccone	Fondazione IRCCS Istituto Neurologico Carlo Besta, Milano, Italy

Ana Gorostidi	Neuroscience Area, Biodonostia Health Research Insitute, San Sebastian, Gipuzkoa, Spain
Caroline Graff	Department of Geriatric Medicine, Karolinska University Hospital-Huddinge, Stockholm, Sweden
Caroline Greaves	Dementia Research Centre, Department of Neurodegenerative Disease, UCL Institute of Neurology, Queen Square, London, UK
Rita Guerreiro	Dementia Research Institute, Department of Neurodegenerative Disease, UCL Institute of Neurology, Queen Square, London, UK
Carolin Heller	Dementia Research Centre, Department of Neurodegenerative Disease, UCL Institute of Neurology, Queen Square, London, UK
Tobias Hoegen	Neurologische Klinik, Ludwig-Maximilians-Universität München, Munich, Germany
Begoña Indakoetxea	Cognitive Disorders Unit, Department of Neurology, Donostia University Hospital, San Sebastian, Gipuzkoa, Spain; Neuroscience Area, Biodonostia Health Research Insitute, San Sebastian, Gipuzkoa, Spain
Vesna Jelic	Division of Clinical Geriatrics, Karolinska Institutet, Stockholm, Sweden
Hans-Otto Karnath	Division of Neuropsychology, Hertie-Institute for Clinical Brain Research and Center of Neurology, University of Tübingen, Tübingen, Germany
Ron Keren	The University Health Network, Toronto Rehabilitation Institute, Toronto, Canada
Robert Laforce	Clinique Interdisciplinaire de Mémoire, Département des Sciences Neurologiques, CHU de Québec, and Faculté de Médecine, Université Laval, Quebec, Canada
Maria João Leitão	Centre of Neurosciences and Cell Biology, Universidade de Coimbra, Coimbra, Portugal
Johannes Levin	Department of Neurology, University Hospital Ulm, Ulm, Germany
Albert Lladó	Alzheimer's disease and Other Cognitive Disorders Unit, Neurology Service, Hospital Clínic, Barcelona, Spain
Sandra Loosli	Neurologische Klinik, Ludwig-Maximilians-Universität München, Munich, Germany
Carolina Maruta	Laboratory of Language Research, Centro de Estudos Egas Moniz, Faculty of Medicine, University of Lisbon, Lisbon, Portugal
Mario Masellis	Sunnybrook Health Sciences Centre, Sunnybrook Research Institute, University of Toronto, Toronto, Canada
Simon Mead	MRC Prion Unit, Department of Neurodegenerative Disease, UCL Institute of Neurology, Queen Square, London, UK
Gabriel Miltenberger	Faculty of Medicine, University of Lisbon, Lisbon, Portugal
Rick van Minkelen	Department of Clinical Genetics, Erasmus Medical Center, Rotterdam, Netherlands
Sara Mitchell	Sunnybrook Health Sciences Centre, Sunnybrook Research Institute, University of Toronto, Toronto, Canada
Katrina Moore	Dementia Research Centre, Department of Neurodegenerative Disease, UCL Institute of Neurology, Queen Square, London UK
Fermin Moreno	Cognitive Disorders Unit, Department of Neurology, Donostia University Hospital, San Sebastian, Gipuzkoa, Spain

Jennifer Nicholas	Department of Medical Statistics, London School of Hygiene and Tropical Medicine, London, UK
Linn Öijerstedt	Department of Geriatric Medicine, Karolinska University Hospital-Huddinge, Stockholm, Sweden
Markus Otto	Istituto di Ricovero e Cura a Carattere Scientifico (IRCCS) Istituto Centro San Giovanni di Dio Fatebenefratelli, Brescia, Italy
Sebastian Ourselin	School of Biomedical Engineering & Imaging Sciences, King's College London, London, UK.
Alessandro Padovani	Centre for Neurodegenerative Disorders, Neurology Unit, Department of Clinical and Experimental Sciences, University of Brescia, Brescia, Italy
Georgia Peakman	Department of Neurodegenerative Disease, UCL Institute of Neurology, UK
Yolande Pijnenburg	Amsterdam University Medical Centre, Amsterdam VUmc, Amsterdam, Netherlands
Cristina Polito	Department of Biomedical, Experimental and Clinical Sciences “Mario Serio”, Nuclear Medicine Unit, University of Florence, Florence, Italy
Sara Prioni	Fondazione IRCCS Istituto Neurologico Carlo Besta, Milano, Italy
Catharina Prix	Neurologische Klinik, Ludwig-Maximilians-Universität München, Munich, Germany
Rosa Rademakers	Department of Neurosciences, Mayo Clinic, Jacksonville, Florida, USA
Veronica Redaelli	Fondazione IRCCS Istituto Neurologico Carlo Besta, Milano, Italy
Tim Rittman	Department of Clinical Neurosciences, University of Cambridge, Cambridge, UK
Ekaterina Rogaeva	Tanz Centre for Research in Neurodegenerative Diseases, University of Toronto, Toronto, Canada
Pedro Rosa-Neto	Translational Neuroimaging Laboratory, McGill Centre for Studies in Aging, McGill University, Montreal, Québec, Canada
Giacomina Rossi	Fondazione IRCCS Istituto Neurologico Carlo Besta, Milano, Italy
Martin Rosser	Dementia Research Centre, Department of Neurodegenerative Disease, UCL Institute of Neurology, Queen Square, London, UK
James Rowe	Department of Clinical Neurosciences, University of Cambridge, Cambridge, UK
Isabel Santana	Neurology Department, Centro Hospitalar e Universitário de Coimbra, Coimbra, Portugal
Beatriz Santiago	Neurology Department, Centro Hospitalar e Universitario de Coimbra, Coimbra, Portugal
Elio Scarpini	Fondazione IRCCS Ca’ Granda Ospedale Maggiore Policlinico, Neurodegenerative Diseases Unit, Milan, Italy; University of Milan, Centro Dino Ferrari, Milan, Italy
Sonja Schönecker	Neurologische Klinik, Ludwig-Maximilians-Universität München, Munich, Germany
Elisa Semler	Department of Neurology, University of Ulm, Ulm
Rachelle Shafei	Dementia Research Centre, Department of Neurodegenerative Disease, UCL Institute of Neurology, Queen Square, London, UK
Christen Shoesmith	Department of Clinical Neurological Sciences, University of Western Ontario, London, Ontario, Canada
Matthis Synofzik	Department of Neurodegenerative Diseases, Hertie-Institute for Clinical Brain Research and Center of Neurology, University of Tübingen, Tübingen, Germany

Miguel Tábuas-Pereira	Neurology Department, Centro Hospitalar e Universitario de Coimbra, Coimbra, Portugal
Fabrizio Tagliavini	Fondazione Istituto di Ricovero e Cura a Carattere Scientifico Istituto Neurologico Carlo Besta, Milan, Italy
Carmela Tartaglia	Tanz Centre for Research in Neurodegenerative Diseases, University of Toronto, Toronto, Canada
Mikel Tainta	Neuroscience Area, Biodonostia Health Research Insitute, San Sebastian, Gipuzkoa, Spain
Ricardo Taipa	Neuropathology Unit and Department of Neurology, Centro Hospitalar do Porto - Hospital de Santo António, Oporto, Portugal
David Tang-Wai	The University Health Network, Krembil Research Institute, Toronto, Canada
David L Thomas	Neuroimaging Analysis Centre, Department of Brain Repair and Rehabilitation, UCL Institute of Neurology, Queen Square, London, UK
Hakan Thonberg	Center for Alzheimer Research, Division of Neurogeriatrics, Karolinska Institutet, Stockholm, Sweden
Carolyn Timberlake	Department of Clinical Neurosciences, University of Cambridge, Cambridge, UK
Pietro Tiraboschi	Fondazione IRCCS Istituto Neurologico Carlo Besta, Milano, Italy
Emily Todd	Department of Neurodegenerative Disease, UCL Institute of Neurology, UK
Philip Vandamme	Neurology Service, University Hospitals Leuven, Belgium; Laboratory for Neurobiology, VIB-KU Leuven Centre for Brain Research, Leuven, Belgium
Rik Vandenberghe	Laboratory for Cognitive Neurology, Department of Neurosciences, KU Leuven, Leuven, Belgium
Mathieu Vandenbulcke	Geriatric Psychiatry Service, University Hospitals Leuven, Belgium; Neuropsychiatry, Department of Neurosciences, KU Leuven, Leuven, Belgium
Michele Veldsman	Nuffield Department of Clinical Neurosciences, Medical Sciences Division, University of Oxford, Oxford, UK
Ana Verdelho	Department of Neurosciences and Mental Health, Centro Hospitalar Lisboa Norte - Hospital de Santa Maria & Faculty of Medicine, University of Lisbon, Lisbon, Portugal
Jorge Villanua	OSATEK, University of Donostia, San Sebastian, Gipuzkoa, Spain
Jason Warren	Dementia Research Centre, Department of Neurodegenerative Disease, UCL Institute of Neurology, Queen Square, London, UK
Carlo Wilke	Department of Neurodegenerative Diseases, Hertie-Institute for Clinical Brain Research and Center of Neurology, University of Tübingen, Tübingen, Germany; Center for Neurodegenerative Diseases (DZNE), Tübingen, Germany
Ione Woollacott	Dementia Research Centre, Department of Neurodegenerative Disease, UCL Institute of Neurology, Queen Square, London, UK
Elisabeth Wlasich	Neurologische Klinik, Ludwig-Maximilians-Universität München, Munich, Germany
Henrik Zetterberg	Dementia Research Institute, Department of Neurodegenerative Disease, UCL Institute of Neurology, Queen Square, London, UK
Miren Zulaica	Neuroscience Area, Biodonostia Health Research Insitute, San Sebastian, Gipuzkoa, Spain
